# Audiological follow-up in a risk-based newborn hearing screening programme: An exploratory study of the influencing factors

**DOI:** 10.4102/sajcd.v65i1.587

**Published:** 2018-10-25

**Authors:** Amisha Kanji, Kirsten Krabbenhoft

**Affiliations:** 1 Department of Speech Pathology and Audiology, University of the Witwatersrand, South Africa

## Abstract

**Background:**

Follow-up return rate in Early Hearing Detection and Intervention (EHDI) programmes is of specific importance as it ensures that benchmarks are met and that no child with suspected hearing loss is left unidentified.

**Objectives:**

The aim of this study was to determine the factors influencing audiological follow-up of high-risk infants in a risk-based newborn hearing screening programme.

**Method:**

A non-experimental, exploratory, qualitative research design was employed. Purposive sampling was used. The study was conducted at a secondary level hospital in the public health care sector in South Africa. Participants comprised 10 caregivers (age range 26–40 years) of infants who had been enrolled in a risk-based newborn hearing screening programme, and returned for follow-up appointments. Data were collected using semi-structured interviews. Responses were recorded by the researcher and a colleague to ensure rigour and trustworthiness of findings. Data were analysed using thematic analysis for open-ended questions and descriptive statistics for the closed-ended questions.

**Results:**

The most common positive contributors that facilitated participants’ attendance at follow-up appointments were: having friendly audiologists; a clear line of communication between caregiver and audiologist and a reminder of the appointment. The most significant perceived challenge that participants described in returning for the follow-up appointment was living in far proximity from the hospital.

**Conclusion:**

Findings of the study revealed that influencing factors on follow-up return rate are demographic, socio-economic, and interpersonal in nature and further suggested the need for an all-inclusive appointment day. It may be of importance to not only look at what is being done to improve the follow-up return rate but also *how* it should be done in terms of professional-to-patient communication and interactions.

## Introduction

The ability of a developing child to hear is of great importance. The age at identification of hearing loss and intervention is vital in ensuring early diagnosis and intervention, as this leads to positive developmental outcomes (Yoshinaga-Itano, 2003). Early detection of hearing loss can be addressed through Universal Newborn Hearing Screening (UNHS) programmes as recommended by the Joint Committee on Infant Hearing (JCIH, 2007) and the Health Professions Council of South Africa (HPCSA, 2007). Because of the fact that Early Hearing Detection and Intervention (EHDI) is not yet fully implemented within the South African context, risk-based newborn hearing screening (screening of newborns and infants with risk factors for hearing impairment) has been recommended as the interim solution where UNHS is not immediately feasible (HPCSA, 2007; Kanji & Khoza-Shangase, 2016).

It has been reported that one of the main challenges for EHDI systems globally is poor follow-up return rate (HPCSA, 2007). Developing countries experience a significantly lower follow-up return rate than developed countries. This is displayed by comparing findings from a developing context to that of a developed context. A study conducted in the United States of America (USA) (developed country) revealed a follow-up return rate of 58% and 100% at two different hospitals (Todd, 2006), whilst studies conducted in Lagos, Nigeria and South Africa (developing countries) revealed return rates of 16% and 31.4%, respectively (Kanji, Khoza-Shangase, & Ballot, 2010; Olusanya, Wirz, & Luxon, 2008). It is understandable that in developing contexts such as South Africa, follow-up return rates are worse than in developed contexts, possibly because of socio-economic factors; an increasing burden of disease; and under-resourced and exhausted public health care services. However, the problem of poor follow-up return rates is still prominent in both developing and developed contexts and has consequences for the appropriate and timely diagnosis of and intervention for hearing impairment.

Follow-up return rates contribute towards the efficacy of EHDI programmes, as follow-up is necessary in order for audiologists to diagnostically assess and confirm the presence of a hearing loss, to evaluate candidacy for various amplification devices and/or assistive technology, and to ensure prompt referral to early intervention services (HPCSA, 2018). Follow-up return rates of 70% or higher are considered ideal (HPCSA, 2018). To improve such efficacy and success of EHDI programmes, various research studies have been conducted that explore the reasons for loss to follow-up. Reasons for loss to follow-up in developing countries, such as Malaysia, include factors, such as lack of communication between parents and screening personnel regarding the need for a follow-up, weakness in the protocol for provision of follow-up appointments, lack of public awareness of childhood hearing loss and the importance of early intervention, accessibility to services, and not having the screening as an integral part of the childcare system (Mukari, Tan, & Abdullah, 2006). Factors related to communication are particularly important within the South African context as it is a multilingual and multicultural context, whereby language differences and mismatches between the audiologist and the caregiver may play a role (Watermeyer, Kanji, & Cohen, 2012). Other studies in Nigeria have revealed cultural beliefs, stigma and place of birth and/or delivery as the possible key determinants of poor compliance to follow-up after discharge (Olusanya, 2009; Olusanya & Akinyemi, 2009). Results from a recent South African study revealed that the most frequent reasons for follow-up default were most commonly related to costs, lack of caregiver knowledge of newborn hearing screening (NHS), caregivers forgetting to bring their infant for the follow-up appointment, lack of caregiver knowledge of results from the initial hearing screening and caregivers being unaware of the recommended follow-up rescreen (Scheepers, Swanepoel, & le Roux, 2014). It was concluded that reminders and increased communication with caregivers are required to decrease follow-up default (Scheepers et al., 2014).

The current study aimed to describe factors influencing caregivers returning for follow-up audiological services in a risk-based NHS programme. Adequate follow-up needs to be ensured for newborns and infants at risk for hearing impairment, as it may not necessarily be detected immediately after birth and the early identification of late or progressive hearing impairment needs to be considered. The current study differs from most studies on follow-up return rates in hearing screening programmes within a developing context that appear to focus on reasons for loss to follow-up, instead of the positive factors that have influenced attendance at follow-up appointments. Conducting research into both the positive factors and perceived challenges may result in a more balanced perspective on follow-up return rate in NHS programmes in South Africa. The current study, therefore, served to investigate the influencing factors of caregivers returning for follow-up audiological services and aimed to focus not only on the perceived challenges, but also the positive influencing factors in an effort to determine if there are factors that may be built on and strengthened.

## Methods

### Objectives

The main aim of this study was to describe the factors influencing audiological follow-up of high-risk infants in a risk-based NHS programme. Specific sub-aims were to:

- describe the reasons for attending audiological follow-up appointments within a risk-based NHS programme;
- determine the perceived challenges faced by caregivers when returning for audiological follow-up within a risk-based hearing screening programme.

### Research design

This study adopted a non-experimental, exploratory, qualitative research design.

### Research context

The study was conducted at a secondary level hospital. At the time of the study, NHS was being conducted on a referral basis from paediatricians and neonatologists and was not routinely conducted. A UNHS feasibility study was also conducted by a paediatrician at this hospital, the results of which indicated that UNHS was not feasible at this site.

### Sampling

A non-probability, purposive sampling method was used.

### Participants

Participants consisted of 10 caregivers of high-risk infants who had undergone two hearing screenings and had been booked for a diagnostic audiological follow-up. The first hearing screening was performed during hospital stay (before discharge) and follow-up appointments were scheduled on days coinciding with the neonatal follow-up clinic with paediatricians (usually 4–6 weeks post discharge). Because of the unavailability of diagnostic audiological equipment at the hospital during the time of the study, diagnostic follow-up was conducted at the nearest university (approximately 6 km – 8 km from the hospital). The caregivers were of infants who underwent hearing screening (within a risk-based NHS programme) at a secondary level hospital in the public health care sector. Participants had to be caregivers of infants who had been enrolled in a risk-based NHS programme and needed to be able to speak and understand English, as either their first or subsequent language, as the researcher did not have access to a trained interpreter at the time.

Participants who attended were all above the age of 20 years. The mean age of participants was 30.5 years (range: 26–40 years; s.d.: 4.79). In terms of the level of education of the participants, all but two participants had a secondary level education. The home languages of participants varied. Two participants’ home language was Sesotho, two participants had isiZulu as the home language, three had English, one had Sepedi, one had Seswati and one had Amharic as the home language.

### Data collection procedures

Data were collected in the form of semi-structured interviews with caregivers who had returned for the follow-up audiological assessment of their infant. Reasons for non-attendance were explored in another larger study (Kanji & Khoza-Shangase, 2018). The interview included both open and closed-ended questions. Both these types of questions were aimed at enquiring about the positive factors, as well as perceived challenges in order to facilitate trustworthiness and rigour. The questions were related to: (1) the reasons why caregivers attended follow-up audiological assessments, (2) the challenges that caregivers faced in attending the follow-up appointment and (3) the changes that caregivers would make to the hearing screening programme or process to reduce the challenges that they faced. The interviews in this study were to be audio recorded but the research site did not permit this, for reasons not specified. Therefore, the researcher scribed and transcribed each interview with a colleague, and these transcriptions were then compared to ensure trustworthiness. A pilot study was conducted prior to the commencement of the current study to ensure the appropriateness of interview questions. Findings from the pilot study indicated the need to simplify the language used which was then implemented.

### Data analysis

Data obtained were analysed using thematic analysis for the open-ended questions and descriptive statistics (measures of frequency) for the closed-ended questions. Thematic analysis allows for themes or topics to be extracted from the data set that is later used as a method of summarising raw data, comparing the frequency of relative themes and looking for co-occurring themes (Namey, Guest, Thairu, & Johnson, 2008).

### Ethical consideration

Ethical clearance was granted by the university’s Human Research Medical Ethics Committee (approved protocol number: M150147). Permission to collect data in the form of interviews was obtained from the Head of the Audiology Department and the Chief Executive Officer (CEO) of the hospital where the research was conducted. Written informed consent was obtained from the participants who were interviewed. The participants were reminded of their right to withdraw from the study at any time. Confidentiality and anonymity of data were maintained by using participant codes instead of names.

## Results and discussion

Findings of this study will be presented and discussed in accordance with the sub-aims of the study. Results pertaining to the related closed-ended questions will be discussed first, followed by the themes emerging from the open-ended questions.

**FIGURE 1:**
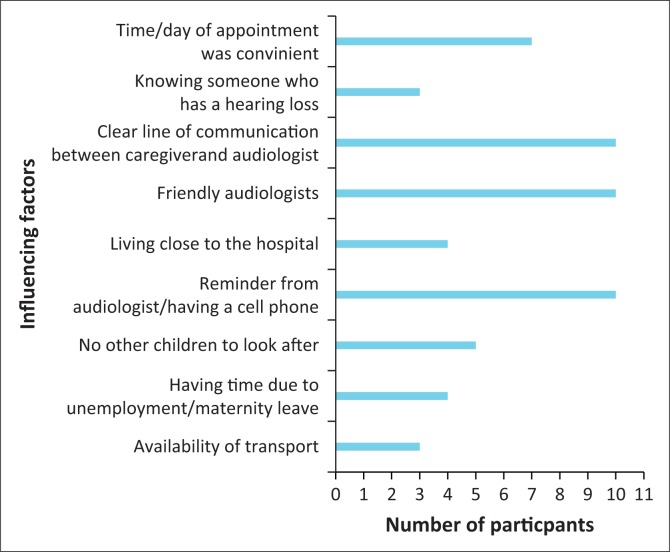
Positive factors influencing follow-up.

The first sub-aim of this study was to obtain information regarding the reasons caregivers attended audiological follow-up appointments. Results from the closed-ended questions related to this sub-aim revealed that the most common positive contributors that facilitated participant’s attendance at follow-up appointments were the following: having friendly audiologists (*n* = 10), a clear line of communication between caregiver and audiologist (*n* = 10) and a reminder from the audiologist or having set one’s own reminder (*n* = 10) (Figure 1).

Of the 10 participants, 7 reported that the time and day of the appointment made it easier to return for the follow-up appointments and five said that not having other children made it easier. The appointments were generally in the morning, at a time and on a day that was suitable for caregivers. Participants, who had other children to look after, reported the morning time as the best time for the appointment as the other children were at school. The other five caregivers, with a primiparous child, stated that it did not matter what time of the day the appointment was, as long as it was not late in the afternoon where they would be likely to get stuck in traffic on the way home. These findings are supported by a study on follow-up return rates, which revealed that failure to follow-up or a delay in follow-up was significant in families with more than two children at home (ASHA, 2008). Thus, highlighting the importance of consideration of the family context.

Four of the 10 caregivers reported that living close to the hospital and having the time because of unemployment or maternity leave were positive influencing factors for them to return for the follow-up appointment. Geographical distance from the hospital and busy work schedules have been shown to be contributing factors to caregivers failing to return for follow-up appointments in NHS programmes (Kanji et al., 2010; Mukari et al., 2006). Therefore, caregivers who do not face these challenges appear to be more likely to return for follow-up hearing screenings. Three out of 10 caregivers felt that knowing someone with a hearing loss and availability of transport were positive influencing factors for returning for follow-up (Figure 1). Exposure to individuals with hearing loss may facilitate awareness of hearing loss and the effects it has on the person, which may understandably increase the likeliness of caregivers returning for follow-up audiological assessments. These findings are supported by literature that has indicated that a lack of caregiver knowledge about NHS and hearing loss was attributed to failure to return for follow-up screenings (Scheepers et al., 2014). With regard to the availability of transport, caregivers interviewed in the current study had all been discharged from hospital and required transport to attend the follow-up appointment at the stipulated facility. Literature has indicated that the need for transport to return for second stage hearing screening, once mother and baby have been discharged from the hospital of birth may contribute to the failure to follow-up (Shulman et al., 2010). The current study’s findings are in support of this literature as it highlights the influence of socio-demographic and contextual factors. These findings are further supported by literature that suggests that factors such as maternal age, ethnicity, maternal education, distance from health care facilities and parity play a role in the uptake of health care services in developing countries (Babalola & Fatusi, 2009; Say & Raineb, 2007; Tsawe et al., 2015) and suggests the need to view follow-up return rate within a broader context.

### Theme 1: The earlier the better

Participants were asked about what facilitated their decision to attend the follow-up appointment. Responses to this open-ended question suggested themes of *the earlier, the better* and *child comes first*. One participant said that the reason she returned for the follow-up appointment was because she wanted:

‘to know about the growing up and hearing [of her child]’. (Participant 2, 37 years, female)

Another caregiver said:

‘… because I want to see my child healthy and everything in her body must be functioning one hundred percent. (Participant 7, 27 years, female)

Two of the participants also remarked that:

‘… if there is a problem, she must get help early’ (Participant 7, 27 years, female)

‘… if there is something wrong they will pick it up earlier, better safe than sorry’. (Participant 8, 28 years, female)

A study by Meintjes and Van Belkum (2013) revealed knowledge deficits amongst caregivers with all developmental categories, with the highest deficit in the domains of speech-language development. This was despite the majority of caregivers indicating that developmental problems should be treated early, as per the awareness demonstrated by participants in the current study. Similarly, Scheepers et al. (2014) in their study reported that one of the most common reasons that caregivers reported not returning for follow-up audiological assessment was associated with a lack of caregiver knowledge in the domain of hearing. Contrary to the current study’s findings, their study found that the majority of caregivers believed that NHS was unnecessary (Scheepers et al., 2014). A study by Swanepoel and Almec (2008) on the other hand revealed that 99% of mothers expressed the desire to have their infant’s hearing screened after birth (Swanepoel & Almec, 2008).

### Theme 2: Child comes first

One participant reported that they returned for the follow-up appointment:

‘… because it’s very important and I know she comes first.’ (Participant 3, 26 years, female)

Another caregiver said:

‘… nothing actually helped me, I knew I needed to be here to make sure everything is fine with her.’ (Participant 4, 31 years, female)

This finding appears to illustrate an instinctual sense of responsibility and accountability of the caregiver towards the child’s health care needs. A child’s development is influenced by the caregiver’s competency to provide care (Meintjes & Van Belkum, 2013). Health care professionals rely on caregivers’ knowledge about the health and development of their children in order to facilitate decision-making, intervention and necessary referrals. Programmes designed to improve the health, development and well-being of children depend on involvement from caregivers (Meintjes & Van Belkum, 2013). Apart from the importance and concern for their child’s development, findings may also be suggestive of a level of commitment from caregivers. Cavalcanti and Guerra (2012) believe that follow-up return rates for screening or diagnostic assessment depict parental commitment more accurately than the initial screening in hospital which only requires maternal consent.

The second sub-aim of the current study was to determine the perceived challenges faced by caregivers when returning for audiological follow-up within a risk-based NHS programme.

**FIGURE 2:**
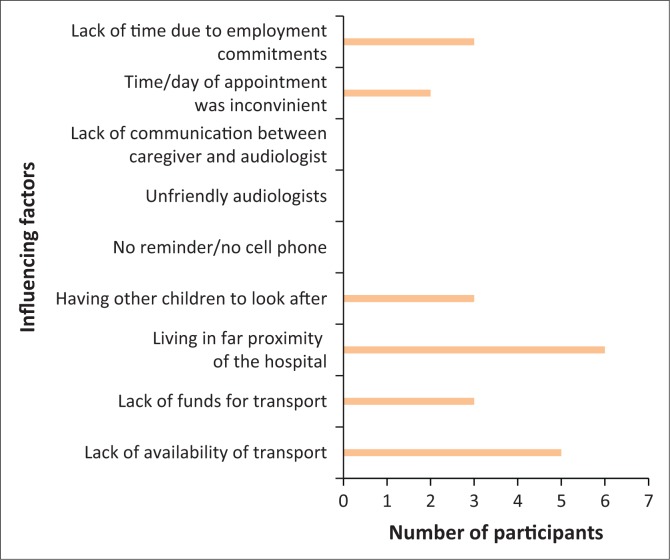
Perceived challenges influencing caregiver’s follow-up.

Responses from the closed-ended questions revealed that the most significant challenge that participants described in returning for the follow-up appointment was living in far proximity from the hospital (*n* = 5). Half the participants felt that lack of availability of transport was a perceived challenge to returning for follow-up appointments. Three out of 10 participants felt that lack of time because of employment commitments, having other children to look after and lack of funds for transport were perceived challenges. The least significant challenge that the participants faced was that time and/or day of appointment was inconvenient (*n* = 2) (Figure 2).

### Theme 3: Lost

When asked an open-ended question about the perceived challenges that they faced, the theme of being ‘lost’ was evident. Participants 2 and 5 stated that ‘not knowing the place’ was a challenge because:

‘… directions and even with directions, it was easy to get lost because taxi’s don’t always drop one at the gate of the place that one needs to be.’ (Participant 2, 37 years, female).

In addition, it is a further challenge to try and navigate around an unfamiliar place by foot. Another participant said that:

‘I took the taxi early, but I was still late here.’ (Participant 8, 28 years, female)

When asked what changes they would make to the process of hearing screening, one participant stated she would prefer if everything took place there, as:

‘… the hospital is the nearest place.’ (Participant 7, 40 years, female)

Another participant said:

‘… only if you could visit the local clinics because they are nearby.’ (Participant 6, 29 years, female)

This finding illustrates feelings of uncertainty and feeling lost as a result of the proximity to and unfamiliarity with the facility. A study conducted in the USA, revealed that families who were compliant with follow-up revealed that a further traveling distance could be a barrier to follow-up (MacNeil, Liu, Stone, & Farrell, 2007). Another study conducted in Brazil, a developing context, revealed through analysis of socio-economic and demographic factors that mothers who did not return to complete the hearing screening generally came from lower-income families who lived in rural areas outside of the city where the NHS services were not being provided (Griz, Merces, Menezes, & Lima, 2009). In exploration of failure to return for follow-up, a study in Malaysia revealed that geographical distance was one of the reasons that caregivers defaulted in returning for second stage hearing screening (Mukari et al., 2006).

These studies outline the fact that in both a developing and a developed context, proximity to resources appears to be a challenge for follow-up. It has been shown that proximity to health care is an important factor affecting various health outcomes (Tanser, Gijsbertsen, & Herbst, 2006). Thus, improving geographical access to primary health care could have a direct impact on the improvement of health outcomes such as hearing loss (Tanser et al., 2006). A retrospective record review study, conducted in South Africa, to determine the follow-up rate for a hearing screening programme implemented as part of a very low birth weight project revealed that efforts to improve the return rate should be concentrated on and strengthened (Kanji et al., 2010). Recommendations to improve return rate included the suggestion that follow-up screening should take place at immunisation clinics that are more accessible to patients (Kanji et al., 2010). This strengthens the theme of proximity and familiarity with a health care facility being a challenge and allows a solution to the challenges of proximity to health care resources, as well as lack of availability of transport, and lack of funds for transport. Having hearing screening take place at immunisation clinics suggests the need for a ‘medical home’ which was also derived from interviews in this study.

### Theme 4: Need for a medical home

Another theme that emerged was the need for a medical home. Two participants stated that if they were to make any changes to the process of hearing screening programmes, they would have ‘… it all in one place’ to avoid having to travel further. It was also reported that, ‘… if everything for the child took place at a hospital nearby’, then the participant could walk to get there. This finding illustrates the need for all child health care services to be provided at the same health care facility and for this facility to be in close proximity for the caregivers. These services include hearing screening at immunisation clinics and/or neonatal follow-up (NNFU) clinics at the hospital of birth. If all of these services could be provided at primary health care clinics (equipped with necessary screening equipment), which are more frequently situated in most areas, this would essentially solve the problems of lack of availability of transport and lack of funds for transport contributing to default to follow-up in hearing screening programmes. Primary health care clinics have been proposed as a platform for NHS with the rationale that it provides an opportunity to reach the entire population resulting in increased coverage of screened infants and improved follow-up return rates (HPCSA, 2007; Swanepoel, Hugo, & Louw, 2006). A systematic community-based NHS programme initiated at eight PHC clinics in the Western Cape was evaluated against the guidelines in the HPCSA (2007) position statement. Although follow-up return rates varied amongst clinics, they were considered to be good (Friderichs, Swanepoel, & Hall, 2012). These findings highlight the advantage of provision of services at the primary health care level, which would facilitate easier accessibility to services by caregivers.

### Theme 5: All at once

The theme of ‘all at once’ also emanated when participants asked what they would change to facilitate the perceived challenges. One participant stated that:

‘I would prefer if the six -month visit was here on the same day as neonatal follow-up.’ (Participant 7, 40 years, female)

Another stated that she would prefer if the screening could take place:

‘… on my appointment day’. (Participant 5, 33 years, female)

This finding illustrates the challenge of trying to schedule appointments when caregivers have limited time because of employment commitments, and/or duties involving taking care of several children. These are challenges that caregivers reported to have faced when returning for follow-up audiological screening. Two different studies have shown that difficulties in scheduling appointments for follow-up hearing screenings have created barriers for families in terms of completing neonatal/infant hearing screening programmes (Harrison, Roush, & Wallace, 2003; MacNeil et al., 2007).

It has been recommended that follow-up hearing screening takes place on the same day as NNFU clinics to improve follow-up return rates (Kanji et al., 2010). This strengthens the results from the current study that revealed that participants would like an ‘all-inclusive’ appointment day where their child will be able to receive more than one health care service. Therefore, having multiple appointments on the same day could potentially eliminate the challenges mentioned above and increase follow-up return rates in EHDI programmes. These findings are supported by Ng, Hui, Lam, Goh and Yeung (2004), who suggest that scheduling of appointments on a single day may also be less costly for caregivers.

A summary of themes and supportive quotes is presented in Table 1.

**TABLE 1:** Summary of themes from qualitative analysis.

Theme	Direct quotes
The earlier the better	‘…want to know about the growing up and hearing.’(Participant 2, 37 years, female) ‘… because I want to see my child healthy and everything in her body must be functioning one hundred percent. If there is a problem, she must get help early.’(Participant 7, 40 years, female) ‘… if there is something wrong they will pick it up earlier, better safe than sorry.’(Participant 8, 28 years, female)
Child comes first	‘… because it’s very important and I know she comes first’ (Participant 3, 26 years, female) ‘… nothing actually helped me, I knew I needed to be here to make sure everything is fine with her.’(Participant 4, 31 years, female)
Lost	‘… not knowing the place’ (Participant 2, 37 years, female) ‘… even with directions, it was easy to get lost because taxi’s don’t always drop at the gate of the place that you need to be.’(Participant 5, 33 years, female) ‘I took the taxi early, but I was still late here’ (Participant 8, 28 years, female) ‘… only if you could visit the local clinics because they are nearby.’(Participant 8, 28 years, female) ‘… the hospital is the nearest place’ (Participant 7, 40 years, female)
Need for a medical home	‘… having it all in one place.’(Participant 4, 31 years, female) ‘… if everything for the child took place at the hospital nearby.’ (Participant 6, 29 years, female)
All at once	‘I would prefer if the 6-month visit was here on the same day as neonatal follow-up.’ (Participant 7, 40 years, female) ‘… on my appointment day.’(Participant 5, 33 years, female)

## Limitations of the study

This study was exploratory in nature and consisted of a small sample of participants who returned for follow-up. In addition, the sample was only obtained from one site. The influence of cultural beliefs on follow-up return was not explored in the current study.

## Conclusion

Findings of this study revealed that the perceived challenges related to follow-up return rate are demographic and socio-economic in nature. The most pertinent results from this study have suggested that positive influencing factors are more interpersonal in nature. It may be of importance to not only look at what is being done to improve the follow-up return rate but also how it should be done in terms of professional-to-patient communication and interactions.
